# Elaboration and Validation of Two Predictive Models of Postpartum Traumatic Stress Disorder Risk Formed by Variables Related to the Birth Process: A Retrospective Cohort Study

**DOI:** 10.3390/ijerph18010092

**Published:** 2020-12-24

**Authors:** Antonio Hernández-Martínez, Sergio Martínez-Vazquez, Julián Rodríguez-Almagro, Miguel Delgado-Rodríguez, Juan Miguel Martínez-Galiano

**Affiliations:** 1 Department of Nursing, Physiotherapy and Occupational Therapy, University of Castilla-La Mancha, 13600 Ciudad Real, Spain; Antonio.HMartinez@uclm.es; 2 Nursing Department, University of Jaen, 23071 Jaen, Spain; sergio_sk8_9@hotmail.com (S.M.-V.); jgaliano@ujaen.es (J.M.M.-G.); 3 Division of Preventive Medicine and Public Health, University of Jaén, 23071 Jaén, Spain; mdelgado@ujaen.es; 4 Consortium for Biomedical Research in Epidemiology and Public Health (CIBERESP), 28029 Madrid, Spain

**Keywords:** post-traumatic stress disorder, predictive model, validation

## Abstract

This study aimed to develop and validate two predictive models of postpartum post-traumatic stress disorder (PTSD) risk using a retrospective cohort study of women who gave birth between 2018 and 2019 in Spain. The predictive models were developed using a referral cohort of 1752 women (2/3) and were validated on a cohort of 875 women (1/3). The predictive factors in model A were delivery type, skin-to-skin contact, admission of newborn to care unit, presence of a severe tear, type of infant feeding at discharge, postpartum hospital readmission. The area under curve (AUC) of the receiver operating characteristic (ROC) in the referral cohort was 0.70 (95% CI: 0.67–0.74), while in the validation cohort, it was 0.69 (95% CI: 0.63–0.75). The predictive factors in model B were delivery type, admission of newborn to care unit, type of infant feeding at discharge, postpartum hospital readmission, partner support, and the perception of adequate respect from health professionals. The predictive capacity of model B in both the referral cohort and the validation cohort was superior to model A with an AUC-ROC of 0.82 (95% CI: 0.79–0.85) and 0.83 (95% CI: 0.78–0.87), respectively. A predictive model (model B) formed by clinical variables and the perception of partner support and appropriate treatment by health professionals had a good predictive capacity in both the referral and validation cohorts. This model is preferred over the model (model A) that was formed exclusively by clinical variables.

## 1. Introduction

Post-traumatic stress disorder (PTSD) has been described as “the complex somatic, cognitive, affective, and behavioral effects of psychological trauma” [1]. PTSD affects the newborn, the mother–child relationship, and the mother’s health and quality of life [2,3,4]. PTSD prevalence can vary considerably, and in a systematic review including 28 studies the average prevalence was 4.0% in the general perinatal population and 18.5% in women at risk [5].

Several factors have been associated with the development of postpartum PTSD [6]. However, research into the obstetric factors involved in PTSD development is scarce. Most studies focus on the influence of birth type [7,8,9,10,11,12,13,14,15] and newborn variables such as prematurity [16,17], low weight [16], low Apgar scores [16,18], type of newborn feeding [7], and newborn hospitalization [9]. Several studies have recently observed a relationship between certain obstetric practices and the risk of PTSD [2,15].

One of the biggest challenges in health is creating tools for predicting the risk of particular health problems. The purpose of these tools is the early identification of those most susceptible to developing the problem or anticipating the appearance of the first symptoms. Several published prediction models exist for PTSD after childbirth, using different variables and populations [9,18,19,20,21,22,23,24,25,26,27]. Although several models exist, only one studied the prediction capacity with ROC curves (19), and none were validated in populations other than those used to create the model. No prediction tool has been made based on the factors related to either the birth process or obstetric practices, and that can be easily used as a screening tool by health professionals. One of the difficulties in creating these prediction models is the choice of initial predictive factors, which may be purely objective or also subjective. We consider that predictors of an objective clinical nature, such as the clinical data of problems and interventions during pregnancy and childbirth, are easily obtainable but may be insufficient to predict complex phenomena such as PTSD. Conversely, the use of variables of a more subjective nature such as anxiety, emotions, depression, among others, although used in various predictive models [19,20,22,23,24,26], is more complex to use. Many of these subjective variables are assessed through scales and questionnaires, some of them long and complex, limiting their application in clinical practice.

Therefore, the objective of this study was to develop and validate two postpartum PTSD risk prediction models, one based exclusively on objective clinical predictive factors and another including subjective factors such as perceptions of partner support and treatment received by professionals.

## 2. Materials and Methods

An observational study was conducted using a retrospective cohort of women who gave birth between 2018 and 2019. The Research Ethics Committee of the province of Jaen approved this study with reference number TD-VCDEPP-2019/1417-N-19. The main tool employed to collect the relevant data for this study was medical records. The women were required to read an information sheet about the study and its objectives and check a box in which they showed their consent to participate in it; that is, they signed an ad hoc digital informed consent. The STROBE statement has been followed in the reporting of this study. [28]

### 2.1. Design and Participants

This analytical and observational study used a retrospective cohort of women who gave birth between 2018 and 2019. A total of 1752 women were included in the referral cohort for the predictive models, which were subsequently validated with a cohort of 875 women. The Clinical Research Ethics Committees of Universidad de Jaen gave ethical approval prior to the start of the study. All of the participants received written information on the study, including the fact that participation was completely voluntary and anonymous.

We used the maximum modeling principle to estimate the sample size. We needed 10 events (women at risk of PTSD) per each incorporated variable [29]. If we consider that our initial model may contain 15 variables, we would need 150 women at risk of PTSD. Taking into account that the prevalence of PTSD in other Spanish studies is reported as 10.6% [2], we would need a minimum of 1415 women for the derivation cohort and at least half as many for validation, about 708 women. Nevertheless, the researchers’ team opted to recruit the maximum number of women.

### 2.2. Data Collection and Information Sources

The main tool employed to collect the relevant data for this study was medical records. The primary outcome variable—risk of PTSD—and the following objective independent variables were collected from the medical records.

Independent variables were:

1. Maternal: maternal age, education level, nationality, attendance at maternal education classes, and the use of a birth plan.
2. Obstetric: previous cesarean section (CS), number of deliveries, induction of labor, type of labor, use of regional analgesia, use of general anesthesia, use of natural analgesic methods, episiotomy, and perineal tear.
3. Fetal: prematurity, twin pregnancy, breastfeeding in the first hour, skin-to-skin contact, admission of the newborn to care unit, type of feeding at hospital discharge.
4. Subjective variables evaluated with a Likert-type scale (scores 1–5): degree of support from the partner during pregnancy, delivery, and postpartum; respectful treatment by professionals during pregnancy, delivery, and postpartum. The different categories used for each variable are detailed in Table 1.

The primary outcome variable, risk of PTSD, was determined using the modified Perinatal Post-Traumatic Stress Disorder Questionnaire (PPQ) [30] (Appendix A: Spanish version). The PPQ is a 14-item measure assessing post-traumatic symptoms related to the childbirth experience, including intrusiveness or re-experiencing, avoidance behaviors, and hyperarousal or numbing of responsiveness. The PPQ also contains one item about feelings of guilt. Response options were modified from the original dichotomous scale to a five-level Likert scale (scored 0 to 4). The total possible score on the modified PPQ ranged from 0 to 56. In the current study, internal consistency was higher than in previous investigations using the dichotomous scaling, with an α = 0.90 [30].

### 2.3. Statistical Analysis

A descriptive statistical analysis using absolute and relative frequencies for qualitative variables and means and standard deviation (SD) for quantitative variables was performed.

The analysis of potential predictive factors, which have been previously identified in the literature as risk factors of delayed onset breastfeeding, was carried out in a bivariate analysis using the chi-square and Student’s *t*-test to estimate qualitative and quantitative variables, respectively. Of these variables, and following Lemeshow’s statistical criteria, associations with *p*-values < 0.25 were selected for inclusion in the multivariate binary logistic regression model [31,32] (Table 1). These analyses were performed in the derivation cohort.

Then, two models were created (Table 2): model A based on exclusively clinical criteria and model B based on clinical criteria plus maternal perceptions of the degree of partner support and the treatment received by healthcare professionals. These models were constructed using backward elimination (RV in SPSS) with the derivation of cohort women’s data. To assess the prediction qualitatively, we used Swets’s criteria, which uses the following category values: 0.5–0.6 (bad), 0.6–0.7 (poor), 0.7–0.8 (satisfactory), 0.8–0.9 (good), and 0.9–1.0 (excellent) [33]. In addition, the Nagerlkerkes R-square and the calibration were determined using the Hosmer–Lemeshow test *p*-value of both models.

The derivation and validation cohorts were compared after using chi-square and Student’s *t*-test for qualitative and quantitative variables, respectively (Table 3). Finally, the AUC-ROC in the validation cohort was estimated for the predictive model that we created (Table 2). In this case, the probabilities used proceed from applying the predictive model created with the derivation cohort using the data of the women in the validation cohort. SPSS 20.0. (SPSS Inc., Chicago, IL, USA) was used for all statistical analyses.

## 3. Results

### Characteristics of Participants

The derivation cohort consisted of 1752 women and the validation cohort 875 women, with a prevalence of PTSD risk of 14.2% (248) and 10.9% (95), respectively. First, we built predictive models using the derivation cohort. The variables associated with the risk of PTSD (screening criterion *p*-value < 0.25) selected for the multivariate analysis were: maternal age, parity, live birth, place of delivery, induced delivery, use of natural methods for pain, regional analgesia, general anesthesia, type of delivery, perineal tear, skin-to-skin contact, breastfeeding the first hour of life, admission of the newborn to care unit, hospital stay, breastfeeding at discharge, postpartum surgical intervention, postpartum readmission, degree of partner support during pregnancy, delivery and postpartum, and degree of respect received from professionals during pregnancy, delivery and postpartum. (Table 1).

Then, two predictive models were created. Model A was based exclusively on clinical variables, and model B consisted of clinical variables plus subjective variables on support received from their partner and treatment received from healthcare professionals (see Table 2). The variables to be included in the final predictive models were selected automatically by the SPSS program, through backward step instruction.

**Figure 1:**
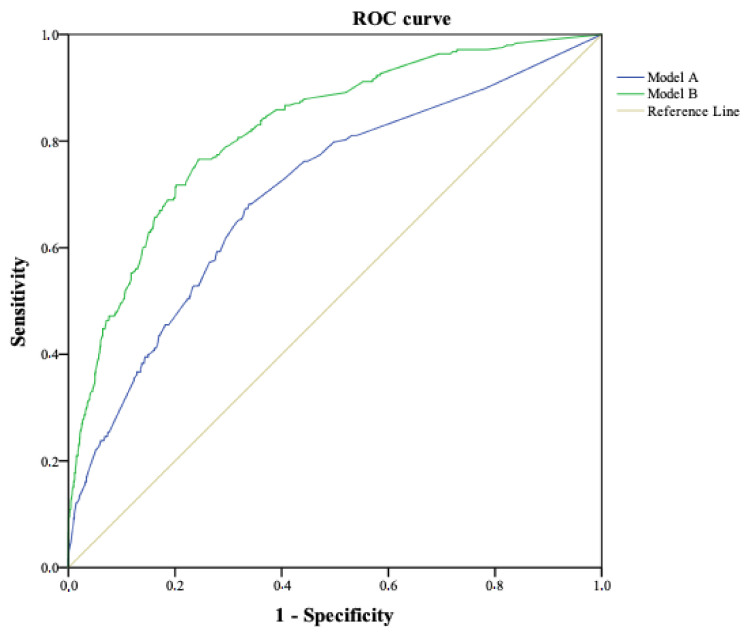
Predictive capacity of model A and model B in the derivation cohort. Area under the ROC curve to determine the predictive ability of the model in the validation cohort, representing the sensitivity on the *y* axis and 1- specificity in the *x* axis.

**Figure 2:**
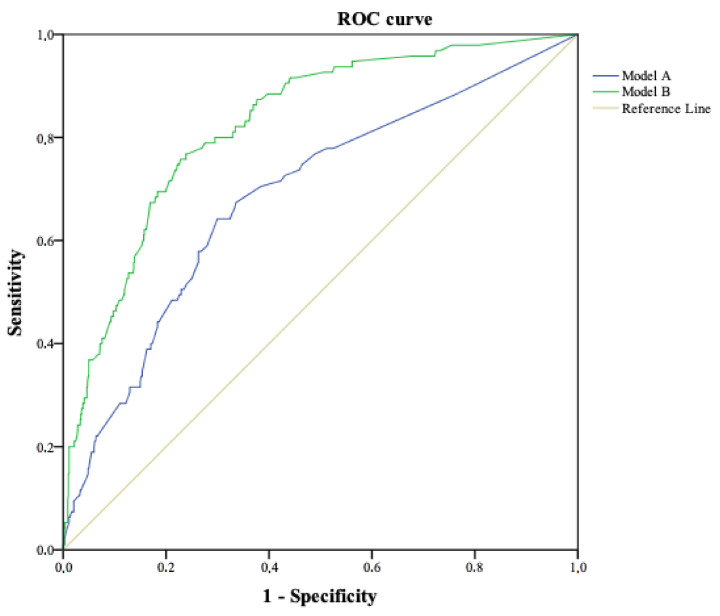
Predictive capacity of model A and model B in the validation cohort. Area under the ROC curve to determine the predictive ability of the model in the validation cohort, representing the sensitivity on the *y* axis and 1-specificity on the *x* axis.

When performing the multivariate analysis, model A included the following variables: type of delivery, skin-to-skin contact, admission of the newborn to care unit, perineal tear, type of infant feeding at discharge, and postpartum hospital readmission. The predictive capacity (AUC-ROC) in the referral cohort was 0.70 (95% CI: 0.67–0.74) (Figure 1), while in the validation cohort it was 0.69 (95% CI: 0.63–0.75) (Figure 2), which is considered as satisfactory in Swets’s criteria.

The predictive factors in the final model B were: type of delivery, admission of the newborn to care unit, type of infant feeding at discharge, postpartum hospital readmission, support received by the partner, and the perception of respect from healthcare professionals. The predictive capacity (AUC-ROC) in the derivation cohort was 0.82 (95% CI: 0.79–0.85) (Figure 1), while in the validation cohort it was 0.83 (95% CI: 0.78–0.87) (Figure 2). This predictive capacity is considered good per Swets’s criteria. Finally, we examined comparability issues in both cohorts, and found no statistically significant differences with any variable except for the risk of PTSD (*p* = 0.018), which was 14.2% (248) in the referral cohort and 10.9% (95) in the validation cohort (see Table 3).

## 4. Discussion

This study presents the main results of the development of two postpartum PTSD risk prediction models. Model A, constructed using only clinical variables, presented a satisfactory predictive capacity (AUC-ROC = 0.70), while model B, constructed with clinical variables and subjective patient perceptions, presented a good predictive capacity (AUC-ROC = 0.82). The predictive variables common to both models were: type of delivery, admission of the newborn to care unit, type of infant feeding at discharge, and postpartum hospital readmission. However, model A also included skin-to-skin contact and the presence of a severe tear as exclusive variables. In contrast, model B included variables relating to the support received from the partner and the perception of respect from healthcare professionals during childbirth.

Currently, there are several studies published with PTSD risk prediction models [9,19,20,21,22,23,24,25,26,27]. However, only the study by Van Heumen et al. studied the prediction capacity with ROC curves [19], presenting an AUC-ROC of 0.795, lower than our best model. Moreover, none have been validated in populations other than those used to create the model, which is an important limitation. The sample sizes were also all smaller than ours, and only one exceeded 1000 subjects [19], and only three studies exceeded 500 subjects [9,19,20]. Some of these studies have been carried out on very specific population groups, such as the study by López et al. [21], who used a sample of women who had a cesarean delivery, excluding most women who give birth vaginally. Other models have included other scales and assessments based on questionnaires of anxiety, emotions, depression, among others, as predictive factors [19,20,22,23,24,26]. The use of multiple questionnaires and scales could hinder their application due to the complexity in obtaining this information and does not allow universal use because these scales and questionnaires were designed and validated to be used in specific populations. In terms of obstetric predictors, only three authors have included this type of clinical variable. Concerning these models, we agree on the inclusion of the variable “type of delivery” as a predictive factor of PTSD risk [9,18,23]; specifically, instrumental delivery [9] and emergency cesarean section [9,18] as they present forms of childbirth with the greatest risks. Although multiple studies associate the type of delivery with the risk of PTSD [7,8,9,10,11,12,13,14,15], and the presence of perineal tears [13,18,34], no predictive models have been developed that include these.

Regarding neonatal variables as factors that influence the risk of PTSD, various variables related to the newborn were identified, including the newborn’s hospital admission [9]. Along similar lines, the risk was also related to the lack of skin-to-skin contact and formula feeding. Although these variables have not been included in other models, they have been related to an increased risk of PTSD in other studies [2,7,15,35]. Although these three variables are related to each other, the authors believe that they also have a partially independent effect. In the first place, not all hospitalized children stop skin-to-skin contact, as in many cases, admission occurs several hours after birth. Second, many women whose children are hospitalized continue to breastfeed despite the great obstacle it poses. The predictive model of Fairtbrother et al. [18] also includes low birth Apgar scores as a factor. In our sample, this variable was not assessed.

Another variable included in our model B was the perception of respectful treatment by healthcare professionals toward women. This aspect is closely related to the concept of obstetric violence and has not been evaluated in other predictive models, despite the existence of publications that identify a relationship between the treatment received by healthcare professionals during childbirth care and the presence of PTSD [36,37]. This aspect takes on particular relevance as the World Health Organization [38] and the United Nations [39,40] report an upward trend in women who perceive inadequate treatment during childbirth care.

Finally, the support provided by the partner plays a relevant role in the risk of PTSD, in such a way that women who perceived that their partners supported them during pregnancy, childbirth and the postpartum period had a lower risk of PTSD, coinciding with the model of Czarnocka and Slade based on a study carried out with 264 women [27].

### Strengths and Limits

One of the potential limitations of this study was that the observed prevalence of PTSD risk was high compared to other studies. In a systematic review with a meta-analysis carried out by Yildiz et al., average rates of 4.0% were found overall (95% CI: 2.77–5.71), and 18.5% (95% CI: 10.6–30.38) in women at risk [5]. The higher prevalence of our sample can be attributed to the use of a screening tool (PPQ) as we did not diagnose PTSD; instead, the risk of presenting PTSD was estimated.

Another limitation of the study was that it was carried out in a population residing in Spain, and even though the validation results were good, they need validation in other countries and cultural contexts. Regarding strengths, in addition to satisfactory predictive capacity, these models have other positive characteristics, such as including only five variables (parsimony principle), using variables that are usually recorded in medical records, and having a justified relationship with the risk of PTSD. We should also highlight that the model was validated in a population different than the one used to create the models, and they also had different prevalences for PTSD risk. These differences are interesting for the extrapolation of results; this validation cohort could almost be considered external validation. Additionally, creating two predictive models may be useful for clinicians because it will expand application possibilities. For example, in situations where verbal contact with patients is not possible, model A, based on clinical variables recorded in medical records, could be used. While when verbal contact with the patient is possible, model B would be the instrument of choice. In particular, this tool is especially useful for professionals who have initial contact with women after childbirth. These professionals can use this tool as screening to identify patients who require further evaluation by more specialized professionals in the field of postpartum PTSD, such as psychologists and psychiatrists.

## 5. Conclusions

In short, two predictive models formed by clinical variables and perceptions of support from their partner and the care received from health professionals presented adequate predictive capacities to predict the risk of postpartum PTSD both in the referral cohort and in the validation cohort. The model of choice includes the woman’s perceptions of support received from her partner and the relationship with healthcare professionals. These models can help identify women at increased risk for postpartum PTSD, increasing the early detection of this increasingly prevalent problem. On the other hand, they can also be useful in primary prevention if health policies are applied that reduce risk factors such as cesarean delivery and inadequate treatment by health professionals and encourage other factors such as skin-to-skin contact and breastfeeding.

## Figures and Tables

**Table 1:** Bivariate analysis of potential predictive factors of post-traumatic stress disorder (PTSD) risk.

Predictor	PTSD (Score PPQ) Derivation Cohort		*p*-Value
	<19 Points *n* (%)	≥19 Points *n* (%)	
**Maternal age**			0.199
35 years	638 (84.6)	116 (15.4)	
>35 years	886 (86.8)	132 (13.2)	
**Education level**			0.401
Primary school	22 (75.9)	7 (24.1)	
Secondary school	77 (87.5)	11 (12.5)	
High school	323 (85.0)	57 (15.0)	
University	1082 (86.2)	173 (13.8)	
**Nationality**			0.986
Spanish	1443 (85.8)	238 (14.2)	
Other	61 (85.9)	10 (14.1)	
**Parity**			**<0.001**
Primiparous	1004 (83.0)	205 (17.0)	
Multiparous	499 (92.1)	43 (7.9)	
**Live newborn**			0.056
No	8 (66.7)	4 (33.3)	
Yes	1496 (86.0)	244 (14.0)	
**Twin pregnancy**			0.319
No	1471 (86.0)	240 (14.0)	
Yes	33 (80.5)	8 (19.5)	
**Previous cesarean section**			**<0.001**
No	1102 (89.7)	126 (10.3)	
Yes	402 (76.7)	122 (23.3)	
**Place of birth**			0.099
Public hospital	1210 (86.2)	194 (13.8)	
Private hospital	262 (83.2)	53 (16.8)	
Midwife-led hospital	6 (85.7)	1 (14.3)	
Home	26 (100.0)	0 (0.0)	
**Labor induction**			**0.007**
No	913 (87.7)	128 (12.3)	
Yes	591 (83.1)	120 (16.9)	
**Regional analgesia**			**0.001**
No	442 (90.4)	47 (9.6)	
Yes	1062 (84.1)	201 (15.9)	
**General anesthesia**			**<0.001**
No	1461 (86.5)	228 (13.5)	
Yes	43 (68.3)	20 (31.7)	
**Natural analgesia**			**0.139**
No	1214 (85.3)	210 (14.7)	
Yes	290 (88.4)	38 (11.6)	
**Type of birth**			**<0.001**
Normal vaginal delivery	912 (91.6)	84 (8.4)	
Instrumental	273 (84.0)	52 (16.0)	
Elective CS	109 (84.5)	20 (15.5)	
Emergency CS	210 (69.5)	92 (30.5)	
**Episiotomy**			0.925
No	1069 (85.8)	177 (14.2)	
Yes	435 (86.0)	71 (14.0)	
**Perineal tear**			**<0.001**
No	940 (83.9)	180 (16.1)	
Mild	512 (90.9)	51 (9.1)	
Severe (III–IV)	52 (75.4)	17 (24.6)	
**Prematurity**			**0.023**
No	1141 (86.4)	223 (13.6)	
Yes	93 (78.8)	25 (21.2)	
**Maternal antenatal classes**			0.123
No	295 (87.5)	42 (12.5)	
Yes (less than 5 classes)	208 (81.9)	46 (18.1)	
Yes (more than 5 classes)	1001 (86.2)	160 (13.8)	
**Breastfeeding 1 h after childbirth**			**<0.001**
No	338 (76.0)	107 (24.0)	
Yes	1166 (89.2)	141 (10.8)	
**Skin-to-skin contact**			**<0.001**
No	302 (73.1)	111 (26.9)	
Yes	1202 (89.8)	137 (10.2)	
**Birth plan**			**<0.001**
No	803 (87.8)	112 (12.2)	
Yes, but not respected	164 (65.3)	87 (34.7)	
Yes, and was respected	537 (91.6)	49 (8.4)	
**Admission of the newborn to care unit**			**<0.001**
No	1324 (87.6)	187 (12.4)	
Yes	180 (74.7)	61 (25.3)	
**Hospital length of stay**			**<0.001**
1 day	122 (91.0)	12 (9.0)	
2 day	779 (90.2)	85 (9.8)	
3 day	365 (82.4)	78 (17.6)	
4 days or more	238 (76.5)	73 (23.5)	
**Infant feeding on discharge**			**<0.001**
Maternal	1226 (88.2)	164 (11.8)	
Mixed	233 (78.5)	64 (21.5)	
Artificial	45 (69.2)	20 (30.8)	
**Postpartum surgical intervention**			**0.001**
No	1449 (86.5)	227 (13.5)	
Yes	55 (72.4)	21 (27.6)	
**Hospital readmission**			**<0.001**
No	1474 (86.4)	232 (13.6)	
Yes	30 (65.2)	16 (34.8)	
	**Mean (SD)**	**Mean (SD)**	
Perception of adequate treatment by health professionals during pregnancy, childbirth and the puerperium. Likert scale 1–5	3.4 (0.93)	2.88 (1.28)	<0.001 *
Perception of support by the couple during pregnancy, childbirth and the puerperium. Likert scale 1–5	2.99 (0.97)	1.67 (1.22)	<0.001 *

Bold: statistically significant differences. * Student–Fisher *t*-test. PPQ: Perinatal Post-Traumatic Stress Disorder Questionnaire.

**Table 2:** Predictive models of PTSD risk during the postpartum.

Model Properties	Model A			Model B		
**Number of Events in Derivation Cohort**	248 (14.2%)					
**Number of Events in Validation Cohort**	95 (10.9%)					
**Nagerlkerkes R-Square**	0.127			0.310		
**Hosmer–Lemeshow Test *p*-Value**	0.133			0.732		
**Risk Factor**	Coef * Beta Value	**OR (95% CI)**	***p*-Value**	Coef * Beta Value	**OR (95% CI)**	***p*-Value**
**Type of birth**						
Normal vaginal delivery		1 (Ref)			1 (Ref)	
Instrumental	0.484	**1.62 (1.10–2.41)**	**0.016**	0.344	1.22 (0.81–1.86)	0.344
Elective CS	0.341	1.41 (0.77–2.57)	0.267	0.200	1.22 (0.68–2.18)	0.499
Emergency CS	1.121	**3.07 (1.96–4.80)**	**<0.001**	0.827	**2.29 (1.56–3.35)**	**<0.001**
**Initiate skin-to-skin contact**	−0.428	**0.65 (0.45–0.96)**	**0.028**			
**Admission of the newborn to care unit**						
No		1 (Ref)			1 (Ref)	
Yes	0.452	**1.57 (1.26–2.87)**	**0.015**	0.503	**1.65 (1.12–2.44)**	**0.012**
**Perineal tear**						
No		1 (Ref)				
Type I–II	−0.020	0.98 (0.67–1.44)	0.919			
Type III–IV	0.795	**2.21 (1.17–4.19)**	**0.015**			
**Infant feeding on discharge**						
Maternal		1 (Ref)			1 (Ref)	
Mixed	0.404	**1.50 (1.06–2.12)**	**0.022**	0.122	1.13 (0.77–1.65)	0.530
Artificial	0.740	**2.10 (1.16–3.79)**	**0.014**	0.803	**2.23 (1.13–4.04)**	**0.021**
**Hospital readmission**	0.934	**2.55 (1.30–5.00)**	**0.007**	1.160	**3.19 (1.43–7.11)**	**0.005**
**Partner’s perception of support (Likert scale 1–5)**				−0.234	**0.79 (0.69–0.91)**	**0.001**
**Perception of respect by professionals (Likert scale 1–5)**				−0.863	**0.42 (0.37–0.48)**	**<0.001**
**Constant**	−2.177			0.545		
**AUC-ROC derivation cohort**		**0.70 (0.67–0.74)**	**<0.001**		**0.82 (0.79–0.85)**	**<0.001**
**AUC-ROC validation cohort**		**0.69 (0.63–0.75)**	**<0.001**		**0.83 (0.78–0.87)**	**<0.001**

OR: odds ratio; Bold: statistically significant differences.

**Table 3:** Comparison of characteristics between the derivation and validation cohort.

Characteristics	Derivation Cohort *N* = 1752 *n* (%)	Validation Cohort *N* = 875 *n* (%)	*p*-Value *
**PPQ**			**0.018**
<19	1504 (85.8)	780 (89.1)	
≥19	248 (14.2)	95 (10.9)	
**Maternal age**			0.930
≤35 years	754 (43.0)	375 (42.9)	
>35 years	998 (57.0)	500 (57.1)	
**Education level**			0.478
Primary school	29 (1.7)	9 (28.6)	
Secondary school	88 (5.0)	37 (4.2)	
High school	380 (21.7)	193 (22.1)	
University	1255 (71.6)	636 (72.7)	
**Nationality**			0.351
Spanish	1681 (95.9)	846 (96.7)	
Other	71 (4.1)	29 (3.3)	
**Parity**			0.092
Primiparous	1209 (69.0)	575 (65.8)	
Multiparous	542 (31.0)	299 (34.2)	
**Live newborn**			0.130
No	12 (0.7)	2 (0.2)	
Yes	1740 (86.0)	873 (99.8)	
**Twin pregnancy**			0.333
No	1711 (97.7)	849 (97.0)	
Yes	41 (2.3)	26 (3.0)	
**Previous cesarean section**			0.167
No	1228 (70.1)	636 (72.7)	
Yes	524 (29.9)	239 (27.3)	
**Place of birth**			0.526
Public hospital	1404 (80.1)	697 (79.7)	
Private hospital	315 (18.0)	155 (17.7)	
Midwife-led hospital	7 (0.4)	3 (0.3)	
Home	26 (1.5)	20 (2.3)	
**Labor induction**			0.213
No	1041 (59.4)	542 (61.9)	
Yes	711 (40.6)	333 (38.1)	
**Regional analgesia**			0.413
No	489 (27.9)	231 (26.4)	
Yes	1263 (72.1)	644 (73.6)	
**General anesthesia**			0.404
No	1689 (96.4)	849 (97.0)	
Yes	63 (3.6)	26 (3.0)	
**Natural analgesia**			0.768
No	1424 (81.3)	707 (80.8)	
Yes	328 (18.7)	168 (19.2)	
**Type of birth**			0.152
Normal vaginal delivery	996 (56.8)	536 (61.3)	
Instrumental	325 (18.6)	146 (16.7)	
Elective CS	129 (7.4)	64 (7.3)	
Emergency CS	302 (17.2)	129 (314.7)	
**Episiotomy**			0.965
No	1246 (71.1)	623 (71.2)	
Yes	506 (28.9)	252 (28.8)	
**Perineal tear**			0.157
No	1120 (63.9)	529 (60.5)	
Mild	563 (32.1)	314 (35.9)	
Severe (III–IV)	69 (3.9)	32 (3.7)	
**Prematurity**			0.821
No	1634 (93.3)	814 (93.0)	
Yes	118 (6.7)	61 (7.0)	
**Maternal antenatal classes**			0.133
No	337 (19.2)	185 (21.1)	
Yes (less than 5 classes)	254 (14.5)	104 (11.9)	
Yes (more than 5 classes)	1161 (66.3)	586 (67.0)	
**Breastfeeding 1 h after childbirth**			0.556
No	445 (25.4)	213 (24.3)	
Yes	1307 (74.6)	662 (75.7)	
**Skin-to-skin contact**			0.422
No	413 (23.6)	194 (22.2)	
Yes	1339 (76.4)	681 (77.8)	
**Birth plan**			0.739
No	915 (52.2)	459 (52.5)	
Yes, but not respected	251 (14.3)	116 (13.3)	
Yes, and was respected	586 (33.4)	300 (34.3)	
**Admission of the newborn to care unit**			0.886
No	1511 (86.2)	753 (86.1)	
Yes	241 (13.8)	122 (13.9)	
**Hospital length of stay**			0.987
1 day	134 (7.6)	69 (7.9)	
2 day	864 (49.3)	434 (49.6)	
3 day	443 (25.3)	216 (24.7)	
4 days or more	311 (17.8)	156 (17.8)	
**Infant feeding on discharge**			
Maternal	1390 (79.3)	681 (77.8)	0.563
Mixed	297 (17.0)	163 (18.6)	
Artificial	65 (3.7)	31 (3.5)	
**Postpartum surgical intervention**			
No	1676 (95.7)	844 (96.5)	0.331
Yes	76 (4.3)	31 (3.5)	
**Hospital readmission**			
No	1706 (97.4)	128 (12.3)	0.480
Yes	46 (2.6)	19 (2.2)	
	**Mean (SD)**	**Mean (SD)**	
Perception of adequate treatment by health professionals during pregnancy, childbirth, and the puerperium. Likert scale 1–5	3.33 (1.00)	3.28 (1.04)	0.334
Perception of support by the couple during pregnancy, childbirth, and the puerperium. Likert scale 1–5	2.80 (1.11)	2.83 (1.26)	0.493

* Student–Fisher *t*-test.

## Data Availability

The data presented in this study are available on request from the corresponding author.

## Appendix A

**Table A1:** Modified perinatal post-traumatic-stress questionnaire symptoms (modified PPQ).

Did you have bad dreams of giving birth or of your baby’s hospital stay? Did you have upsetting memories of giving birth or of your baby’s hospital stay? Did you have any sudden feelings as though your baby’s birth was happening again? Did you try to avoid thinking about childbirth or your baby’s hospital stay? Did you avoid doing things that might bring up feelings you had about childbirth or your baby’s hospital stay (e.g., not watching a TV show about babies)? Were you unable to remember parts of your baby’s hospital stay? Did you lose interest in doing things you usually do (e.g., did you lose interest in your work or family)? Did you feel alone and removed from other people (e.g., did you feel like no one understood you)? Did it become more difficult for you to feel tenderness or love with others? Did you have unusual difficulty falling asleep or staying asleep? Were you more irritable or angry with others than usual? Did you have greater difficulties concentrating than before you gave birth? Did you feel more jumpy (e.g., did you feel more sensitive to noise, or more easily startled)? Did you feel more guilt about the childbirth than you felt you should have felt?

* Notes: the response and weight of scores for each question: (0) nothing; (1) once or twice; (2) sometimes; (3) often, but less than 1 month; (4) often for more than a month. The clinical range for high-risk mothers is established at 19 or more points.
